# Whole-Genome Sequence of the Multidrug-Resistant Staphylococcus warneri Strain G1M1F, Isolated from Mice with Mastitis

**DOI:** 10.1128/mra.00275-23

**Published:** 2023-04-24

**Authors:** M. Nazmul Hoque, Zannatara Moyna, Golam Mahbub Faisal, Ziban Chandra Das

**Affiliations:** a Molecular Biology and Bioinformatics Laboratory, Department of Gynaecology, Obstetrics and Reproductive Health, Bangabandhu Sheikh Mujibur Rahman Agricultural University, Gazipur, Bangladesh; University of Rochester School of Medicine and Dentistry

## Abstract

We sequenced the genome of Staphylococcus warneri G1M1F, a multidrug-resistant strain isolated from fecal samples of mice with induced mastitis. The complete genome of G1M1F consists of one chromosome of 2,504,515 bp and two plasmid replicons of 28,679 and 8,615 bp; it comprises 32.7% GC content, with 60× genome coverage.

## ANNOUNCEMENT

Mastitis in lactating mammals is known to be caused by a plethora of opportunistic pathogens of variable origin, where the severity and outcome depend on the environment, pathogens, and the host (1–3). Recent studies have demonstrated that transplantation of fecal and milk microbiota from mastitis-infected cows could induce mastitis in pregnant mice, with microbiome dysbiosis (1, 4). Staphylococcal mastitis is a major and costly problem in dairy cattle (5, 6). Although Staphylococcus aureus has been described as one of the most important mastitis pathogens in cow and buffalo (5, 6), coagulase-negative staphylococci (CoNS), including Staphylococcus chromogenes, S. epidermidis, S. haemolyticus, S. warneri, and S. intermedius, are increasingly being recognized as etiologic agents of mastitis in different countries (7, 8). CoNS species such as *S. warneri* are opportunistic mastitis pathogens (7). Although the role of S. aureus as an etiological agent of bovine mastitis has been elucidated previously (5, 6, 8), the relative importance of *S. warneri* in mastitis pathophysiology has been poorly studied. Moreover, the emergence of multidrug-resistant strains among CoNS species has now become a global public health concern.

Fecal samples from mice in which mastitis had been induced (at our laboratory animal research facility; 24.09°N, 90.41°E) were plated on nutrient agar for overnight incubation at 37°C. Pure colonies were isolated and subsequently streaked on mannitol salt agar (Oxoid, Thermo Scientific, UK) plates and incubated at 37°C for 24 h (9). Phenotypic identification of the isolates was performed based on the colony morphology, Gram staining (Gram-positive cocci), and biochemical tests such as catalase, indole, methyl red, Voges-Proskauer (VP), oxidase, urease, and triple sugar iron tests (9). Isolate G1M1F was confirmed as *S. warneri* by 16S rRNA gene sequencing (9). The multidrug-resistant (resistant to >6 antibiotics) *S. warneri* isolate was incubated in nutrient broth (Biolife, Italy) overnight at 37°C, and DNA was extracted from the harvested culture using the QIAamp DNA minikit (Qiagen, Hilden, Germany). The DNA purity and concentration were checked using the NanoDrop 2000 UV-visible (UV-Vis) spectrophotometer (Thermo Fisher, Waltham, MA, USA). The Nextera DNA Flex library prep kit (Illumina, San Diego, USA) was used to generate libraries from 1 ng DNA, and whole-genome sequencing of the prepared libraries was performed using the Illumina MiSeq sequencer with a 2 × 250-bp protocol. The paired-end raw reads (*n* = 19,001,144) were trimmed using Trimmomatic v0.39 (with the parameters leading:20, slidingwindow:4:20:20, trailing:20, minlen = 36) to remove Illumina adapters, known Illumina artifacts, and phiX reads (10) and quality checked using FastQC v0.11.7 (11). Reads with Phred scores of >20 were assembled using SPAdes v3.9.0 (12), and the quality of the assembled genome was checked using QUAST v5.0.2 (13). The NCBI Prokaryotic Genome Annotation Pipeline (PGAP) v6.4 (14) was used for genome annotation. The ResFinder v4.0 (15), PlasmidFinder (16), VFDB (17), and RAST FIGfam v70 (18) databases were used to predict antimicrobial resistance genes (ARGs), plasmids, virulence factors, and metabolic functions, respectively, in the assembled genome. Default parameters were used for all software unless otherwise specified. This study was reviewed and approved by the Animal Research Ethics Committee (AREC) of the Bangabandhu Sheikh Mujibur Rahman Agricultural University, Bangladesh (reference number FVMAS/AREC/2023/6679; 16 January 2023).

The draft genome of G1M1F had 60× coverage and 32 identified contigs. The annotated chromosome length, GC content, and *N*_50_ value of the assembled genome were 2,504,515 bp, 32.5%, and 147,068 bp, respectively. The lengths of the largest and smallest contigs were 544,053 and 1,601 bp, respectively. The G1M1F genome contained 278 subsystems with 34% coverage, 2,426 protein coding sequences, and 38 RNA genes. Twenty-seven ARGs conferring resistance to multiple antibiotics and metals and 112 virulence factors were detected in the draft genome. Two plasmid replicons, rep5b and repUS35 (28,679 and 8,615 bp, respectively), were identified in the genome (with 95% identity and 60% coverage).

### Data availability.

The whole genome of *S. warneri* G1M1F has been deposited at NCBI/GenBank under the accession number JAQPCE000000000, and the raw reads were deposited under SRA accession number SRR24033088 (BioProject accession number PRJNA929689). The version described in this paper is version JAQPCE010000000.

## References

[1] Ma C, Sun Z, Zeng B, Huang S, Zhao J, Zhang Y, Su X, Xu J, Wei H, Zhang H. 2018. Cow-to-mouse fecal transplantations suggest intestinal microbiome as one cause of mastitis. Microbiome 6:200. doi:10.1186/s40168-018-0578-1. 30409169 PMC6225715

[2] Boix-Amorós A, Hernández-Aguilar MT, Artacho A, Collado MC, Mira A. 2020. Human milk microbiota in sub-acute lactational mastitis induces inflammation and undergoes changes in composition, diversity and load. Sci Rep 10:18521. doi:10.1038/s41598-020-74719-0. 33116172 PMC7595153

[3] Hoque MN, Istiaq A, Clement RA, Sultana M, Crandall KA, Siddiki AZ, Hossain MA. 2019. Metagenomic deep sequencing reveals association of microbiome signature with functional biases in bovine mastitis. Sci Rep 9:13536. doi:10.1038/s41598-019-49468-4. 31537825 PMC6753130

[4] Hoque MN, Rahman MS, Islam T, Sultana M, Crandall KA, Hossain MA. 2022. Induction of mastitis by cow-to-mouse fecal and milk microbiota transplantation causes microbiome dysbiosis and genomic functional perturbation in mice. Anim Microbiome 4:43. doi:10.1186/s42523-022-00193-w. 35794639 PMC9258091

[5] Hoque M, Das Z, Rahman A, Haider M, Islam M. 2018. Molecular characterization of *Staphylococcus aureus* strains in bovine mastitis milk in Bangladesh. Int J Vet Sci Med 6:53–60. doi:10.1016/j.ijvsm.2018.03.008. 30255079 PMC6147393

[6] Hoque MN, Talukder AK, Saha O, Hasan MM, Sultana M, Rahman AA, Das ZC. 2022. Antibiogram and virulence profiling reveals multidrug resistant *Staphylococcus aureus* as the predominant aetiology of subclinical mastitis in riverine buffaloes. Vet Med Sci 8:2631–2645. doi:10.1002/vms3.942. 36136962 PMC9677375

[7] Hosseinzadeh S, Saei HD. 2014. Staphylococcal species associated with bovine mastitis in the north west of Iran: emerging of coagulase-negative staphylococci. Int J Vet Sci Med 2:27–34. doi:10.1016/j.ijvsm.2014.02.001.

[8] Taponen S, Pyörälä S. 2009. Coagulase-negative staphylococci as cause of bovine mastitis—not so different from Staphylococcus aureus? Vet Microbiol 134:29–36. doi:10.1016/j.vetmic.2008.09.011. 18977615

[9] Hoque MN, Istiaq A, Clement RA, Gibson KM, Saha O, Islam OK, Abir RA, Sultana M, Siddiki AZ, Crandall KA, Hossain MA. 2020. Insights into the resistome of bovine clinical mastitis microbiome, a key factor in disease complication. Front Microbiol 11:860. doi:10.3389/fmicb.2020.00860. 32582039 PMC7283587

[10] Bolger AM, Lohse M, Usadel B. 2014. Trimmomatic: a flexible trimmer for Illumina sequence data. Bioinformatics 30:2114–2120. doi:10.1093/bioinformatics/btu170. 24695404 PMC4103590

[11] Andrews S. 2017. FastQC: a quality control tool for high throughput sequence data.

[12] Bankevich A, Nurk S, Antipov D, Gurevich AA, Dvorkin M, Kulikov AS, Lesin VM, Nikolenko SI, Pham S, Prjibelski AD, Pyshkin AV, Sirotkin AV, Vyahhi N, Tesler G, Alekseyev MA, Pevzner PA. 2012. SPAdes: a new genome assembly algorithm and its applications to single-cell sequencing. J Comput Biol 19:455–477. doi:10.1089/cmb.2012.0021. 22506599 PMC3342519

[13] Gurevich A, Saveliev V, Vyahhi N, Tesler G. 2013. QUAST: quality assessment tool for genome assemblies. Bioinformatics 29:1072–1075. doi:10.1093/bioinformatics/btt086. 23422339 PMC3624806

[14] Tatusova T, DiCuccio M, Badretdin A, Chetvernin V, Nawrocki EP, Zaslavsky L, Lomsadze A, Pruitt KD, Borodovsky M, Ostell J. 2016. NCBI Prokaryotic Genome Annotation Pipeline. Nucleic Acids Res 44:6614–6624. doi:10.1093/nar/gkw569. 27342282 PMC5001611

[15] Bortolaia V, Kaas RS, Ruppe E, Roberts MC, Schwarz S, Cattoir V, Philippon A, Allesoe RL, Rebelo AR, Florensa AF, Fagelhauer L, Chakraborty T, Neumann B, Werner G, Bender JK, Stingl K, Nguyen M, Coppens J, Xavier BB, Malhotra-Kumar S, Westh H, Pinholt M, Anjum MF, Duggett NA, Kempf I, Nykäsenoja S, Olkkola S, Wieczorek K, Amaro A, Clemente L, Mossong J, Losch S, Ragimbeau C, Lund O, Aarestrup FM. 2020. ResFinder 4.0 for predictions of phenotypes from genotypes. J Antimicrob Chemother 75:3491–3500. doi:10.1093/jac/dkaa345. 32780112 PMC7662176

[16] Carattoli A, Zankari E, García-Fernández A, Voldby Larsen M, Lund O, Villa L, Møller Aarestrup F, Hasman H. 2014. In silico detection and typing of plasmids using PlasmidFinder and plasmid multilocus sequence typing. Antimicrob Agents Chemother 58:3895–3903. doi:10.1128/AAC.02412-14. 24777092 PMC4068535

[17] Liu B, Zheng D, Jin Q, Chen L, Yang J. 2019. VFDB 2019: a comparative pathogenomic platform with an interactive Web interface. Nucleic Acids Res 47:D687–D692. doi:10.1093/nar/gky1080. 30395255 PMC6324032

[18] Overbeek R, Olson R, Pusch GD, Olsen GJ, Davis JJ, Disz T, Edwards RA, Gerdes S, Parrello B, Shukla M, Vonstein V, Wattam AR, Xia F, Stevens R. 2014. The SEED and the Rapid Annotation of microbial genomes using Subsystems Technology (RAST). Nucleic Acids Res 42:D206–D214. doi:10.1093/nar/gkt1226. 24293654 PMC3965101

